# Issues of Fish Consumption for Cardiovascular Disease Risk Reduction

**DOI:** 10.3390/nu5041081

**Published:** 2013-03-28

**Authors:** Susan K. Raatz, Jeffrey T. Silverstein, Lisa Jahns, Matthew J. Picklo

**Affiliations:** 1 Grand Forks Human Nutrition Research Center, Agricultural Research Service, United States Department of Agriculture, Grand Forks, ND 58201, USA; E-Mails: lisa.jahns@ars.usda.gov (L.J.); matthew.picklo@ars.usda.gov (M.J.P.); 2 Department of Food Science and Nutrition, University of Minnesota, Minneapolis, MN 55108, USA; 3 Office of National Programs, Agricultural Research Service, United States Department of Agriculture, Beltsville, MD 21769, USA; E-Mail: jeff.silverstein@ars.usda.gov; 4 Department of Chemistry, University of North Dakota, Grand Forks, ND 58203, USA

**Keywords:** fish, cardiovascular disease, omega-3 fatty acids, EPA, DHA aquaculture

## Abstract

Increasing fish consumption is recommended for intake of omega-3 (*n*-3) fatty acids and to confer benefits for the risk reduction of cardiovascular disease (CVD). Most Americans are not achieving intake levels that comply with current recommendations. It is the goal of this review to provide an overview of the issues affecting this shortfall of intake. Herein we describe the relationship between fish intake and CVD risk reduction as well as the other nutritional contributions of fish to the diet. Currently recommended intake levels are described and estimates of fish consumption at a food disappearance and individual level are reported. Risk and benefit factors influencing the choice to consume fish are outlined. The multiple factors influencing fish availability from global capture and aquaculture are described as are other pertinent issues of fish nutrition, production, sustainability, and consumption patterns. This review highlights some of the work that needs to be carried out to meet the demand for fish and to positively affect intake levels to meet fish intake recommendations for CVD risk reduction.

## 1. Introduction

Cardiovascular disease (CVD) is a major cause of morbidity and death in the United States and many other countries. Prevention of CVD is a public health goal and comprises several avenues of action, of which one of the most effective may be the inclusion of fish in the diet [1]. Fish intake is related to CVD risk reduction in both observational and clinical intervention trials and the 2010 *Dietary Guidelines for Americans* recommends consumption 8 ounces or more of seafood weekly to provide an average consumption of 1750 mg per week (250 mg per day) of eicosapentaenoic acid (EPA; 20:5*n*-3) and docosahexaenoic acid (DHA; 22:6*n*-3) which are long chain omega-3 (LC*n*-3) fatty acids. Commonly consumed high omega-3 (*n*-3) “oily fish” are salmon, mackerel, sardines, anchovies, trout, and tuna [2].

Table 1 shows a summary of published CVD outcomes related to reported fish intake. Although there are inconsistencies in results between studies, likely based on study method variability, the overwhelming conclusion is that of an association of reduced CVD risk with fish intake, particularly for reduced risk of death from cardiac events. Fish species providing high levels of EPA and DHA may be most protective against CVD. Recent data suggest that docosapentaenoic acid (DPA; 22:5*n*-3), an intermediate product in the conversion of EPA to DHA, also found in marine sources may have its own particular effects upon CVD-related outcomes [3,4,5,6]. In general, LC*n*-3 may reduce CVD risk through anti-lipidemic, anti-inflammatory, anti-platelet, and anti-arrhythmic mechanisms. An in-depth discussion of the biochemical and physiological bases for LC*n*-3 and CVD risk reduction is beyond the scope of this review and the reader is referred to several of many comprehensive reviews [7,8,9,10].

**Table 1:** Reported fish consumption and cardiovascular disease.

Reference	Year	Primary outcomes
Kromhout *et al.* [11] (Zutphen Study)	1985	An inverse relationship was observed between fish consumption and coronary artery disease death over 20 years of follow-up.
Burr *et al.* [12] (DART Study)	1989	Fatty fish intake (≥2–3 times/week) reduced mortality in men after myocardial infarction.
Dolecek [13] (MRFIT Study)	1991	Consumption of small amounts of fish (reported as *n*-3 fatty acids) associated with reduced risk of coronary heart disease.
Siskovick *et al.* [14]	1995	Intake of fatty fish (≥1 mean/week) was associated with a 50% reduction in risk of primary cardiac arrest.
Ascherio *et al.* [15]	1995	No significant relationship was observed between fish intake and risk of coronary disease.
Rodriguez *et al.* [16] (Honolulu Heart Program)	1996	High fish intake (≥2 times/week) among heavy smokers (>30 cigarettes/day) reduced relative risk of coronary heart disease mortality by half.
Daviglus *et al.* [17] (Chicago Western Electric Study)	1997	An inverse relationship was observed between fish intake and coronary heart disease, especially non-sudden death from myocardial infarction.
Albert *et al.* [18] (Physicians Health Study)	1998	Fish intake ≥once weekly associated with reduced sudden cardiac death.
Oomen *et al.* [19] (Seven Countries Study)	2000	Total fish consumption was not associated to coronary heart disease mortality; fatty fish consumption was associated with reduced coronary heart disease mortality.
Iso *et al.* [20] (Nurses’ Health Study)	2001	Higher fish consumption (≥1–3 times/month) associated with reduced risk of thrombotic infarction but not related to hemorrhagic stroke.
Yuan *et al.* [21]	2001	Men consuming ≥200 g of fish/shellfish weekly had reduced risk of fatal MI compared to those consuming <50 g/week; no risk reduction was observed for stroke or ischemic heart disease.
Hu *et al.* [22] (Nurses’ Health Study)	2002	Higher fish consumption (≥1–3 times/month) associated with reduced coronary heart disease risk among women.
He *et al.* [23] (Health Professionals Follow Up Study)	2002	Risk of ischemic stroke was significantly lower in men who ate fish 1–3 times/month.
Hu *et al.* [24] (Nurses’ Health Study)	2003	Higher fish consumption (≥1–3 times/month) associated with reduced coronary heart disease risk among women with diabetes.
Mozaffarian *et al.* [25] (Cardiovascular Health Study)	2003	Consuming tuna or other broiled or baked fish ≥3 times/week reduced risk of ischemic heart disease death; reported fried fish/fish sandwich intake showed no association.
Osler *et al.* [26]	2003	Fish intake of ≥1 time/week compared to <2 times/month was not associated with the incidence of coronary heart disease.
Erkkila *et al.* [27] (Estrogen Replacement and Atherosclerosis Trial)	2004	Consumption of fish (≥2 servings of fish or ≥1 serving of tuna or dark fish weekly) was related to significantly reduced progression of coronary artery stenosis in women with coronary artery disease.
Jarvinen *et al.* [28]	2006	Higher fish consumption was associated with a decreased risk of coronary heart disease in women while no association was observed with men
Streppel *et al.* [29] (Zutphen Study)	2008	Intake of fatty fish was associated with reduced risk of sudden coronary death.
Yamagishi *et al.* [30]	2008	An inverse relationship was observed between fish intake and cardiovascular mortality, especially for heart failure.
De Goede *et al.* [31]	2010	Fish consumption reduced fatal myocardial infarction and coronary heart disease risk in a dose dependant manner; no association was observed with nonfatal myocardial infarction.

Multiple demand-side and supply-side factors shape oily fish consumption. Intake of fish varies due to regional, economic, cultural, and personal factors. On the other hand, there are global production forces that influence availability and price. Widespread increases in the intake of fish to meet recommendations for CVD risk reduction will only be possible if there are adequate fish supplies to satisfy the demand [32]. The supply of seafood from global capture fisheries has plateaued and is unlikely to supply adequate amounts of additional seafood to the world’s growing population [33] and thus aquaculture production is essential to meet the shortfall [34]. The purpose of this review is to provide a discussion of these factors shaping the consumption of oily fish, including current dietary recommendations and intakes, production practices and sustainability, and public awareness [33].

## 2. Intake Recommendations for Fish

### 2.1. National and International Recommendations for Fish Intake

The current *Dietary Guidelines for Americans* 2010 (DGA) recommendation for fish intake is “at least 8 ounces of cooked seafood per week”; and to “select some seafood that is rich in omega-3 fatty acids, such as salmon, trout, sardines, anchovies…” [2]. The American Heart Association (AHA) 2002 recommendation is the consumption of two to 3.5 oz (~200 g) cooked portions of fish, particularly fatty fish high in *n*-3 fatty acids, at least two times weekly for risk reduction of CVD [1]. In individuals with established CVD, the recommendation is for approximately 1 g and EPA and DHA daily, preferably from oily fish. European recommendations for *n*-3 fatty acids and fish, primarily for the prevention on CVD, are approximately 250–500 mg/day through consumption of oily fish [35].

The estimated average intake of LC*n*-3 (EPA, DPA and DHA) for Americans is 60–170 mg/day, mostly through fish consumption [36,37,38,39]. There are no identifiable differences by race or ethnicity in intake patterns, although intakes are highest among the top income tertile [37,38,39]. In the US, no Dietary Reference Intake (DRI) for EPA and DHA currently exists [40]; but based upon recent research, there is strong support in the US for the establishment of a DRI of 250–500 mg/day of EPA and DHA for CVD risk reduction [41,42]. At best, EPA and DHA intake in the US is only 20% of the proposed DRI level.

Fish and seafood are the major food source of LC*n*-3 in the diet however, the content is dependent on the species, habitat, fat content, and in the case of farmed varieties, the source of aquafeed. Table 2 illustrates the *n*-3 content of fish and seafood from the USDA Database for Standard Reference [43]. Enhancement of *n*-3 fatty acids in circulation and tissue from fish intake is dependent on sources rich in long chain *n*-3. Philibert [44] evaluated total fish intake from local freshwater and market sources in a cross-sectional evaluation of participants and determined that only oily fish intake was significantly associated with serum *n*-3 concentrations.

**Table 2:** Omega-3 content of fish and seafood (g/100 g) [43].

Fish	Total omega-3	EPA	DPA	DHA
*Farmed*				
Salmon, Atlantic, farmed	2.359	0.862	0.393	1.104
Trout, rainbow, farmed	0.824	0.217	0.091	0.516
Catfish, channel, farmed	0.089	0.017	0.015	0.057
*Wild*				
Herring, Pacific	1.830	0.969	0.172	0.689
Salmon, Atlantic, wild	1.723	0.321	0.287	1.115
Herring, Atlantic	1.626	0.709	0.055	0.862
Sardine, Pacific, canned in tomato sauce	1.457	0.532	0.061	0.864
Whitefish, mixed species	1.421	0.317	0.163	0.941
Mackerel, canned	1.334	0.434	0.104	0.796
Salmon, pink, canned	1.166	0.334	0.089	0.743
Sardine, Atlantic, canned in oil	0.982	0.473	0.000	0.509
Tuna, white (Albacore), canned in water	0.880	0.233	0.018	0.629
Bass, striped	0.754	0.169	0.000	0.585
Mollusks, oyster, Pacific	0.708	0.438	0.020	0.250
Trout, rainbow, wild	0.693	0.167	0.106	0.420
Sea bass, mixed species	0.671	0.161	0.076	0.434
Salmon, Chinook, smoked (lox), regular	0.523	0.183	0.073	0.267
Catfish, channel, wild	0.464	0.130	0.100	0.234
Mollusks, mussel, blue	0.463	0.188	0.022	0.253
Cisco	0.405	0.095	0.053	0.257
Pike, walleye	0.349	0.086	0.038	0.225
Crustaceans, crab, blue	0.320	0.170	0.000	0.150
Croaker, Atlantic	0.306	0.123	0.086	0.097
Flatfish (Flounder/Sole)	0.273	0.137	0.028	0.108
Crustaceans, crab, Dungeness	0.237	0.219	0.010	0.008
Tuna, light, canned in water	0.228	0.028	0.004	0.196
Halibut, Atlantic and Pacific	0.210	0.066	0.016	0.128
Cod, Atlantic	0.194	0.064	0.010	0.120
Crustaceans, lobster, northern	0.176	0.102	0.006	0.068
Pollock, Alaska	0.169	0.049	0.004	0.116
Tilapia **	0.134	0.005	0.043	0.086
Haddock	0.136	0.042	0.005	0.089
Cod, Pacific	0.134	0.034	0.004	0.096
Mollusks, clams, mixed species	0.114	0.043	0.007	0.064
Mollusks, scallop, mixed species	0.106	0.042	0.003	0.061
Crustaceans, shrimp, mixed species **	0.064	0.030	0.003	0.031

** The majority consumed in the US are of farmed source.

Processing and cooking of fish has the potential to alter the fatty acid content of the consumed product. When salmon is baked to recommended temperatures, the LC*n*-3 content is unaffected and there are likely no alteration in the beneficial effects afforded [45]. 

### 2.2. Current Intake Levels of Fish-Usual Intake

Fish is not a habitually consumed food in the US, making it difficult to estimate actual intake levels. At the national level, disappearance data is used to estimate the edible amount of a commodity available for consumption by adding imports and landings and subtracting exports. Consumption of seafood in the US was estimated to be approximately 15.0 lbs (6.8 kg) in 2011, which is equivalent to nearly 24.2 kg measured as live weight [46]. Per capita seafood consumption varies widely by region and country; the global average is about 18.8 kg live weight equivalent, with Japan reaching over 58 kg [34]. The most popular seafood consumed in the US is shrimp with nearly 2 kg per capita consumed in 2011 followed by canned tuna and salmon. Of the top 10 consumed sea foods (Table 3) in the US, five are either primarily or substantially produced by aquaculture. Shrimp has been the dominant seafood consumed for years with the majority of shrimp imported to the US from farms overseas [47]. Per capita salmon consumption in the US, at 0.9 kg, represents the single largest contributor to dietary intake of LC*n*-3 (see Table 2, Table 3) [48].

**Table 3:** US per capita fish consumption [49].

	2011		2010		2009	
	Species	Lbs	Species	Lbs	Species	Lbs
1	Shrimp	4.2	Shrimp	4.0	Shrimp	4.10
2	Canned Tuna	2.6	Canned Tuna	2.7	Canned Tuna	2.5
3	Salmon	1.952	Salmon	1.999	Salmon	2.04
4	Pollock	1.312	Tilapia	1.450	Pollock	1.454
5	Tilapia	1.287	Pollock	1.192	Tilapia	1.208
6	Pangasius	0.628	Catfish	0.800	Catfish	0.849
7	Catfish	0.559	Crab	0.573	Crab	0.594
8	Crab	0.518	Cod	0.463	Cod	0.419
9	Cod	0.501	Pangasius	0.405	Clams	0.413
10	Clams	0.331	Clams	0.341	Pangasius	0.356
Total All Species		15.0		15.8		15.8

The most comprehensive data on individual fish intake in the US is derived from the population-based What We Eat in America-National Health and Nutrition Examination Survey (WWEIA-NHANES) [38,39]. Tran *et al.* [50] report that per capita mean intake of fish is 8.78 g/day; with 24% from tuna, 19% from salmon, and 11% from breaded fish while shrimp intake accounts for 48% of the total per capita shellfish intake. Unfortunately, not all individuals consume fish. In a recent report using WWEIA-NHANES 1999–2004 data, Tran *et al.* [50] estimate that 69% of individuals are usual fish consumers (defined as having consumed fish at least once in the past month). Tuna is the most frequently eaten (35%) with a mean intake of 5.87 g/day; salmon accounts for 18% of fish consumed (8.8 g/day); and breaded fish is the third most frequently consumed (7.26 g/day). Even among salmon consumers, the weekly intake is only 2 ounces. On a weekly basis, fish consumers eat an estimated 3.3 oz per eating occasion of all fish combined; far less than the 4 oz serving recommended by the DGA [50]. To bring Americans closer to meeting the goal of consuming 4 oz of fatty fish twice a week, public health intervention is needed at three levels: first, to shift the non-fish eaters to adoption of regular fish consumption; second, to more than double the amount of fish consumed; and third, to move consumers to dramatically increase the amount of fatty fish consumed.

### 2.3. Fish *versus n*-3 Supplement Intake

Consumption of fish as a source of LC*n*-3 fatty acids has been demonstrated to be superior to the use of *n*-3 oil supplements, thereby making fish intake the most effective source of LC*n*-3 fatty acids [51]. The level of LC*n*-3 in circulation and tissue stores is higher after intake of fish than of fish oil supplements. Serum levels of EPA and DHA were demonstrated to be higher after the intake of cooked or smoked salmon (129% rise in EPA and 45% rise in DHA) compared with cod liver oil (CLO) supplements (106% and 25%, respectively) for 8 weeks despite the EPA and DHA dosing of 1.2 g/day as fish and 3 g/day from 15 mL CLO [51]. Both salmon and CLO contain EPA and DHA predominantly as triglyceride. However, the enhanced uptake from fish is likely attributable to the physiochemical structure of the lipids in fish tissue which may enhance digestive and absorptive properties [51]. Thus it is evident that consuming fish as the source of LC*n*-3 instead of supplements allows for the intake of a lower volume of EPA and DHA to raise levels in circulation. 

## 3. Nutritional Contribution of Fish

### Fish Consumption Replacing Meat (PUFA *vs.* SFA)

Although the intake of oily fish provides long chain *n*-3 fatty acids there may be benefit from the consumption of all seafood products. The nutrient content of fish varies greatly by species and in individual fish depending on age, sex, environment and season [34]. Factors such as feed intake, migratory swimming and sexual changes related to spawning all affect the nutritional quality of fish. 

Fish are classified as lean, semi-fatty or fatty depending on the total storage of fat in body tissues. Lean fish have a low fat content that is relatively stable while that in oily species varies considerably according to season and geographic region of harvest [34,52]. Fish are uniquely different from other animal protein sources in that they contain up to 40% of the total lipid as highly unsaturated LC fatty acids with freshwater species containing a somewhat lower level than marine sources. Like milk, eggs and meat, fish is high in protein and contains a complete amino acid profile. Most fish contain 15%–20% of total body weight as protein. In addition, all fish are a good source of B vitamins and, in the case of the fatty species Vitamins A and D. All fish are a valuable source of calcium, phosphorus, iron, copper and selenium; with saltwater fish also providing iodine. 

Replacement of other dietary animal products with fish in the diet, whether high in *n*-3 fatty acids or not, may be cardioprotective due to the reduced saturated fatty acid and cholesterol content of these products as well as their high polyunsaturated fatty acid content compared to other animal protein sources. Most fish and seafood are low in cholesterol with the exception of crab, lobster, shrimp and oysters [43]. 

## 4. Fish Quality and Sustainability

### 4.1. Wild Fish Stock

The catch from wild fish stocks is generally considered to be at or near the biological maximum, with approximately 90% of the fish stocks globally rated as fully or overexploited [34]. At present, capture fisheries production is relatively stable at approximately 90 million tons per year since the 1990s. 

### 4.2. Status of Aquaculture Fish World Wide

Atlantic salmon is widely farmed with Norway and Chile being two of the primary countries of production and contributing over 1.5 million tons per year; Canada, United Kingdom, United States, and other countries have major salmon farming efforts as well. Bivalve shellfish (clams, oysters, mussels) are also commonly farmed seafood with global production. These products provide good levels of EPA and DHA (Table 2) [53]. Bivalve shellfish production is expanding worldwide and accounts for approximately 10%–12% of the total seafood consumption globally [34]. Aquaculture production of marine fish species other than salmon with a high content of LC*n*-3, is on a much smaller scale. Seabass, seabream, flounder, and cobia, are a few of the farmed marine species that have been developed over recent years and decades. The interest in farming cod (*Gadus morhua*) was reignited several years ago when the supply of wild cod was scarce and the price rose. In 2009 Norway produced approximately 20,000 tons of farmed cod [54]. However a rebound in the natural cod stocks has made the fish more plentiful and the economics of producing farmed cod became much less attractive. A number of other species are being farmed in varying quantities including flounder (*Paralichthys* sp.), sablefish (*Anoplopoma fimbria*) and cobia (*Rachycentron canadum*) and about 180 additional species.

### 4.3. Effect of Aquaculture Practice on *n*-3 Status of Fish

The LC*n*-3 fatty acids one gets from consuming fish are accumulated in the flesh of the fish as it eats other organisms that contain LC*n*-3. The sources of all dietary LC*n*-3 fatty acids are marine algae; primarily microalgae and plankton. Seafood produced through aquaculture contains LC*n*-3 fatty acids in an amount that reflects that of the feed fed. Generally the target levels of LC*n*-3 in fish produced by aquaculture are intended to match the levels determined analytically in wild caught fish, rather than to meet the dietary requirement of the fish being produced. Determining the dietary requirements of fish for the LC*n*-3 fatty acids is complicated and depends on a number of factors, such as the size, age and reproductive status; furthermore, it is recognized that the requirements for the health of the fish are generally considerably below the target level to provide health benefits to humans. Therefore, fish are fed a diet that provides enough LC*n*-3 for good health of the fish to within a short time before slaughter. In the last month or months before harvest, the fish diet is changed to a higher content of LC*n*-3 to meet the values seen in typical wild fish of a given species so that the LC*n*-3 levels in the final aquaculture product will be similar to a wild product [55]. This practice is sometimes referred to as phase feeding.

#### Alternative Feeds Initiatives

As the total volume of seafood produced by aquaculture increases, the amount of fish meal and fish oil used in aquatic animal feeds has grown considerably. Nevertheless, at the same time the total amount of fish meal and fish oil use has been rising, the fraction of the diet that is made up of fish meal and fish oil is declining [56]. Typical fish feeds have reduced the inclusion rate of fish meal in the diet from near 50% to around 25% of total volume in the past 15 years [56] and fish oil usage has remained relatively constant through the development of phase feeding practices discussed above. As fishmeal usage has declined the use of alternative protein sources has grown considerably and plant based proteins are a major source of protein for aquatic feeds. Other protein and lipid sources are being examined, such as algal meals, insect and other invertebrate animal-meals, and meals of single celled organisms from fermentation processes [57]. Currently soy protein and other plant meals with complementary amino acid profiles are meeting the majority of the demand because of the much greater availability of these plant proteins. The impact of these changes of aquafeed ingredients on the composition of the fish and ultimately the impacts on the health of the consumers have not yet been explored in great detail. There is apparently some capacity for LC*n*-3 production from vegetable oils [58,59,60] in some fish, so this potential remains to be explored and developed.

## 5. Influences on the Public’s Choice to Consume *n*-3-Rich Fish

There are multiple factors that potentially influence the public’s choice to consume *n*-3-rich fish. These choices are affected by individual factors such as taste and convenience [61], demographic factors such as age [62,63,64], cultural background [61,65], socio-educational status [65], economic factors such as affordability and availability [64], knowledge of health benefits from eating *n*-3-rich fish, toxicological concerns such as contamination by mercury and dioxin, and environmental concerns of overfishing and habitat destruction [53,64,66,67,68]. 

### 5.1. Benefits *versus* Risks of Fish Consumption

The public receives mixed messages regarding the health benefits *vs*. the health risks of fish consumption. These messages are derived from scientific research, governmental recommendations and governmental advisories and are often reported on the popular media. The public receives information on the positive effects of fish consumption for CVD risk reduction and the need for DHA for cognitive development in children. However, there has been concern raised of the potential for accumulation of mercury into fatty fish [53,66]. In order to address the concern of mercury, the 2004 joint advisory US Food and Drug Administration and US Environmental Protection Agency recommended that women who might become pregnant, are pregnant or nursing, and young children should eat ≤12 ounces/week (2 meals) of fish such as canned tuna and salmon and avoid fish such as shark and king mackerel [69]. This intake of two servings/week falls within the AHA recommended dietary guidelines for prevention of CVD. There are other concerns owing to the potential for exposure to polychlorinated biphenyls (PCBs) from fish sources. However, as noted by the European Food Safety Authority and others, the risk of exposure to PCBs and related compounds from consumption of fish is no different than that for other meats and dairy products [70]. Recent publications examining epidemiological data have weighed the opposing factors of health benefits *vs*. contaminant exposure [53,66,67,71,72]. These studies are in agreement that the benefits of fish consumption of 2 servings weekly outweigh the potential health risks. 

### 5.2. Health Benefit Awareness

What is the impact of the health benefits *vs*. health risks messaging upon consumer choice and how well does the information from academic analyses reach the general public? Research by Verbeke and colleagues [73] demonstrated that understanding of health risks and health benefits was dependent upon age as younger adults were more cognizant of the health risks of fish consumption while older adults had more awareness of health benefits and perceived fish consumption as healthy. In this same study, it was shown that awareness of health risks was positively associated with education level, a result supported by others [73]. The way in which the conflicting messaging is presented also influences consumer choice. In a study of Belgian consumers, it was shown that positive health message increased consumer consumption of fish whereas, as expected, a negative health message had the opposite effect [63]. Interestingly, when both a negative and positive message was presented, the message presented first in order had greater impact [63]. Other data indicate that too much information becomes confusing and unwanted in some portions of the population [66,74]. Unfortunately, media messages favor reporting health risks of seafood consumption *versus* reporting health benefits. A recent study by Greiner and colleagues demonstrated that US television and newspaper media favored reporting health risks of fish consumption 4 times more than health benefits between 1993 and 2007 [75].

### 5.3. Knowledge of Health Benefits of Fish

Conflicting data exist as to whether fish consumption is related to views of “healthy” eating. In a study of Danish consumers, it was found that consuming fish or fish oil was not strongly related to concepts of “healthy” eating [76]. On the other hand, a study of Norwegian consumers indicated that intake of *n*-3 fatty acids and fish overall was associated with other indices of a healthy diet [62]. This same study also found that intake was greater in individuals >60 years *vs.* individuals <49 years [62]. A study of Pienak and colleagues [77] examined the role of familial CVD history upon *n*-3 rich fish consumption in multiple European countries. The results from this study indicated that history of CVD did not influence fish consumption. Although fish intake was generally accepted as “healthy”, there were country-specific differences in the depth of knowledge of these nutritional issues [77]. 

### 5.4. Farmed *vs.* Wild Fish

Does the perception of farmed *vs*. wild-caught marine fish alter consumer choice? Farmed, Atlantic salmon contains high levels of *n*-3 fatty acids [78] and per portion contains amounts similar to that of wild Atlantic salmon [72,78,79]. Current world-wide demand for fish cannot be met by wild-caught fish and much of the available *n*-3 fish, such as salmon, is farmed. In a study of Belgian consumers, sustainability was an important overall consideration but did not influence the overall frequency of fish consumption [64]. In a subset, the choice to not eat wild fish was related to sustainability whereas the choice to eat wild fish was attributed to the belief of better nutritive value as well as better taste [64]. In a related study, it was observed that consumers in general perceived no differences between farmed and wild fish [79]. However, it was noted that there was a large gap between consumer knowledge of nutrient content of wild *vs*. farmed fish and actual scientific data [80]. The data from these studies suggest that sustainability and the controversy of farmed *vs*. wild caught, while viewed as important, are not major factors in the general public’s choice to eat fish and that other factors (e.g., availability, cultural, health-benefit) have greater influence.

### 5.5. Future Studies

Existing studies of factors impacting consumer choice of fish intake have been mostly in northern European populations *vs.* other populations. There are very few published data examining the influences of geography, age, culture and other demographic characteristics that shape fish intake in the US population and how or if knowledge of health benefits or health concerns play a role. This lack of data is surprising given the cultural and geographical diversity of the US population and the importance of fish consumption for health. 

## 6. Conclusions

There is consistent evidence supporting risk reduction of CVD due to fish consumption, particularly the intake of oily fish high in LC*n*-3. Although both the *DGA* and the AHA recommend fish consumption twice weekly, intake of fish in the United States remains low with few individuals meeting recommended intake levels and others consuming none at all. 

The global capture fish industry is unable to meet demands as fish supplies are already fully exploited and the current world-wide consumption of fish is not met by wild-caught fish. The aquaculture industry is essential to support the demand for fish.

Aquaculture supplies a large percentage of the fish demand at present and the industry will continue to grow to meet increasing needs. Work must be done to develop both marine and non-marine aquaculture practices to meet the demand for fish production. Continued research is required to optimize the feed of farm raised fish, to improve production practices, reduce the need for valuable wild fish stock in feed, and to produce fish that is accepted by consumers and contains the nutrients required to improve human health.

Getting the message of the benefits of fish consumption to consumers is an important endeavor. A public health education program is required to provide the public with the message of fish consumption for health. The first steps will require the elucidation of the forces that shape fish consumption by Americans and other populations followed by efforts to increase fish intake to meet recommendations.
